# EVALUATING THE SENIOR EMPLOYMENT AND SOCIAL ACTIVITY SUPPORT PROGRAM IN KOREA: A SYSTEMATIC REVIEW OF SATISFACTION

**DOI:** 10.1093/geroni/igad104.3310

**Published:** 2023-12-21

**Authors:** Young Bin Koh, Yeonjung Lee

**Affiliations:** Chung-Ang University, Seoul, Republic of Korea; Chung-Ang University, Seoul, Republic of Korea

## Abstract

For older people, jobs provide not only income security, but also a social role. Jobs also give them a sense of subjective security in terms of maintaining self-esteem and self-identity, and improve their overall life satisfaction. The Senior Employment and Social Activity Support Program (SESAP) provides for older Koreans for supplemental income security and social participation in old age by creating and providing social jobs that match the aptitudes and abilities of the senior citizens. It has been 20 years since the SESAP was introduced, and many studies have accumulated to evaluate the program in many different aspects. This study aims to summarize the existing studies focusing on satisfaction with program among participants. A systematic literature review was conducted to identify empirical research studies written in Korean and published in a peer-reviewed journal between 2004 and 2023. To this end, we searched through RISS, KISS, and DBpia databases using the selected keywords and obtained 126 articles. After removing duplicates and excluding gray literature, 26 articles were selected and analyzed through a peer-review process. Analysis of the selected literature shows that a majority of the program participants are women, and satisfaction with salary was lower compared to other types of program satisfaction (i.e., job content, work environment, and relationship with co-workers). Lastly, satisfaction with program are associated with other indicators such as better life satisfaction, and quality of life. This systematic review provides suggestions for further research to improve the satisfaction with SESAP among participants.

